# We can reject a zombie apocalypse

**DOI:** 10.3325/cmj.2021.62.422

**Published:** 2021-08

**Authors:** Charles H. Calisher

Demonstrating that not everything on the US Centers for Disease Control (CDC) and Prevention website (*https://www.cdc.gov/)* is depressing or even serious, on May 16, 2011 the sophisticated Ali Khan, MD, then Director of the CDC Office of Public Health Preparedness and Response and Retired Assistant Surgeon General, now Dean of the College of Public Health and at the University of Nebraska Medical Center in Omaha, Nebraska, posted a blog on that site (1). The posting, entitled “Preparedness 101: Zombie Apocalypse,” was a humorous attempt to communicate with younger people (and Haitians?) regarding the need to prepare for outbreaks and epidemics of diseases. Some very few people missed the point and criticized CDC for spending public funds (if it did, it was not much) on such a ridiculous posting. Of course, those few people neglected to understand that the blog was not actually about an unnatural (and artificial) disaster, it was obviously intended to point out that we all need to prepare for natural disasters and used the word “zombies” as a lure to potential readers; it was successful in this effort. The blog even included a list of necessities for an emergency, including “Water (1 gallon per person per day), food (stock up on non-perishable items that you eat regularly), medications (this includes prescription and non-prescription medicines), tools and supplies (including utility knife, tape, battery powered radio, etc.), sanitation and hygiene supplies (household bleach, soap, towels, etc.), clothing and bedding (a change of clothes for each family member and blankets), important documents (copies of your driver’s license, passport, and birth certificate to name a few), and first aid supplies (Although you’re a goner if a zombie bites you, you can use these supplies to treat basic cuts and lacerations that you might get during a tornado or hurricane)”.

Zombies are mythological, undead, corporeal revenants, people who have returned, especially from the dead, created through the reanimation of a corpse. In movies, shows, and literature, zombies are often depicted as being created (the cause of zombiism?) by an infectious virus, which is passed on via bites and contact with bodily fluids. Noted Harvard University psychiatrist Steven Schlozman wrote a (fictional) medical paper on the zombies presented in the film “Night of the Living Dead” and referred to their condition as Ataxic Neurodegenerative Satiety Deficiency Syndrome caused by an infectious agent; it is great fun to read this serious take on a non-serious issue (2). The Zombie Survival Guide identifies the cause of zombies as a virus called “solanum.” Other zombie origins shown in films include radiation from a destroyed NASA Venus probe (as in “Night of the Living Dead”), as well as mutations of existing conditions such as prions in mad-cow disease or viruses in measles and rabies. Schlozman, nevertheless, suggested that the etiology of zombiism hypothetically might involve “a virus that destroys much of the brain's frontal lobe,” the seat of higher cognition and regulator of our baser impulses. Stripped of this moderating influence, the brain's unchecked amygdala, what Schlozman calls our “crocodile brain,” drives victims into a state of unrelieved rage. Meanwhile, “damage to the cerebellum impedes balance, sending zombies lurching forward in their wide-legged shamble. Damage to the ventromedial hypothalamus blocks any feeling of satiety, “producing a never-ending hunger.” And the moaning? In a word: constipation. An all-brain (not all-bran) diet is too high in protein and entirely void of fiber. Now you know.

All this nonsense is supposed to be fun, invented or encouraged by imaginative writers and film makers. Recently, however, a strange occurrence was observed in Denmark (3). In 2020, Denmark culled millions of farmed American minks (*Neovison vison*) after coronavirus infections were recognized among these animals. In November, Danish authorities announced a plan to cull all farmed mink in the country after nearly 300 farms reported SARS-CoV-2 infections among their animals. The virus had mutated while spreading among the mink, and the Danish authorities worried that the mutant virus might spill over to humans and worsen the pandemic; a few thousand mink escape Danish farms each year, so some infected mink could have ventured into the wild and passed the virus to other animals. In subsequent months, hastily buried mink carcasses began rising from the ground, propelled by the gases seeping from their decomposing flesh, according to news reports, which suggested it was like a scene pulled from a bad zombie movie.

Well, we know that zombies cannot be real entities but it is at least enjoyable to let the mind wander about the possibility that certain historical figures might return from the dead and wander through our front yards or even ring our doorbells, asking for or demanding something to eat, namely us.

As perhaps the richest person to have ever lived, Genghis Khan’s net worth would have amounted to what has been estimated as $US 194 billion. Undoubtedly one of the most successful military leaders of all time, he led the Mongol Empire, which at its height stretched from China to Europe, and controlled the largest contiguous empire in history. No one accumulates that much money without killing at least some people and he killed his share. Coming back as a zombie, and with a long non-life ahead of him, he might have added to his death toll, not worrying about anything but extending his empire. And empires are how wealth is accumulated. Genghis Kahn really did not have money as such, he had land. All the land that exists is all the land that will ever exist, so owning all the land from Mongolia to Vietnam, from Persia to downtown Kiev, would have made him a real estate magnate of considerable means. Jeff Bezos (Amazon) is supposed to have $US 182 billion and is still very much alive, piling it on until he retired last month, but with opportunities to accumulate even more, but Genghis Kahn, Alan the Red, John D. Rockefeller, Andrew Carnegie, Catherine the Great, Joseph Stalin, Empress Wu, Akbar I, Emperor Shenzong, Julius Augustus Caesar, and the King of Timbuktu, Mansa Musa (who was “indescribably rich,” because his kingship ruled over the largest production of gold in the world in the 14th century) are long dead but, while they lived, variously donated money to humanitarian causes, amassed political power, managed to accumulate literally trillions of US$ of wealth but did not have enough cash to buy a cup of coffee, stole from the rich and the poor and otherwise built trade routes, or even owned Egypt for a time (J. A. Caesar). Still, the possibility of a comeback as a zombie likely would have fascinated them.

I am probably safe to assume that Adolph Hitler would have liked to kill more people than he did. Obsessed as he was with death, arising from the ashes of Hell, he probably would have tried to get back in the game. If his victims had such an opportunity (or a vote), I doubt they would take it. Alternatively, I would like to see Thomas Jefferson, Elvis Presley, John Kennedy, Mickey Mantle (a baseball player), and others at least once again, but not if they would be after my brain.

Well, a zombie apocalypse is not going to happen. Therefore, we do not need to prepare for it but at least we can use Dr Ali Khan’s (no relation to Genghis Khan, as far as I know) suggestion to prepare for another pandemic or even just a simple epidemic. His suggestions noted above were intended to inform one individual or a few individuals but once a pandemic virus is loose, much greater effort must be made on a national or international scale.

There is a fictitious tale told in many medical schools: A man is sitting at the side of a river when he notices a human body floating by. He jumps in the river, grabs the body and swims back to the shore. He revives the person but, no sooner does he sit back down than he sees another body floating by. Again, he jumps in and saves the person. He now is growing tired, but when two more bodies come floating past, he heroically saves them. Now he is exhausted and realizes he cannot do more. He thinks to himself, “I cannot continue this. I will walk up river and find out who is throwing these people in the river.”

The moral of this story is that it is better to prevent a problem from occurring than to solve it. So too, predicting, aborting, or, better yet, preventing epidemics is now seen as the best ways for people to protect themselves and each other from ravages such as we have seen following the recognition of the current coronavirus pandemic.

An attempt has been made to try to do this. PREDICT is an epidemiological research program funded by US Agency for International Development grants. The program was represented as an early warning pandemic system. However, predicting events, particularly biologic events, is problematic, as success depends on a large number of variables, some of which are unknown. The work being done by the Wuhan Institute of Virology with coronaviruses, for example, was supposed to do this kind of research but that seems not to have been a raging success, at least to this point. It is now obvious that an international collaborative effort is required, so that all institutions performing such work (ie, looking for hazardous disease agents) do so within a uniform set of rules and regulations and that these procedures are strictly overseen and adhered to, honesty overtaking possible embarrassment. As the current pandemic demonstrates, it is too dangerous to do otherwise. Laboratory safety and security regulations and other aspects of proper handling of test samples should be an absolute requirement and “gain-of-function” research, if done at all, would best be done under strict controls and with viruses for which a vaccine is available or that are sensitive to drugs. Although they never should, infectious agents escape from laboratories from time to time. Mostly, they do little or no harm, but that is by chance. We cannot rely on chance to save us from disaster. A very recent paper proposes just such oversight, some of which has been done or will soon be done (4).

Another problem in accomplishing such controls is, to put it politely, the often-complex relationship between investigators and administrators. If administrators were scientist-administrators that might ease the friction but those are rare individuals; rare but not impossible to find. If all controls were left to people who are strictly administrators, we might never get anything done. (We would have to begin this adventure only if all parties are in agreement about the goal, not about parking spaces.) Meanwhile, for us as individuals, it might be best if we copied Dr Khan’s list and stocked up.

Finally, no matter the country, waiting for young politicians to solve our problems is not a reliable solution. As Oscar Wilde said, “I am not young enough to know everything.”
